# Supplementary figures and images for: Derivation and validation of a predictive model for chronic stress in patients with cardiovascular disease
Source: PLoS One. 2022 Oct 18;17(10):e0275729. doi: 10.1371/journal.pone.0275729 (PMC9578618; doi:10.1371/journal.pone.0275729)

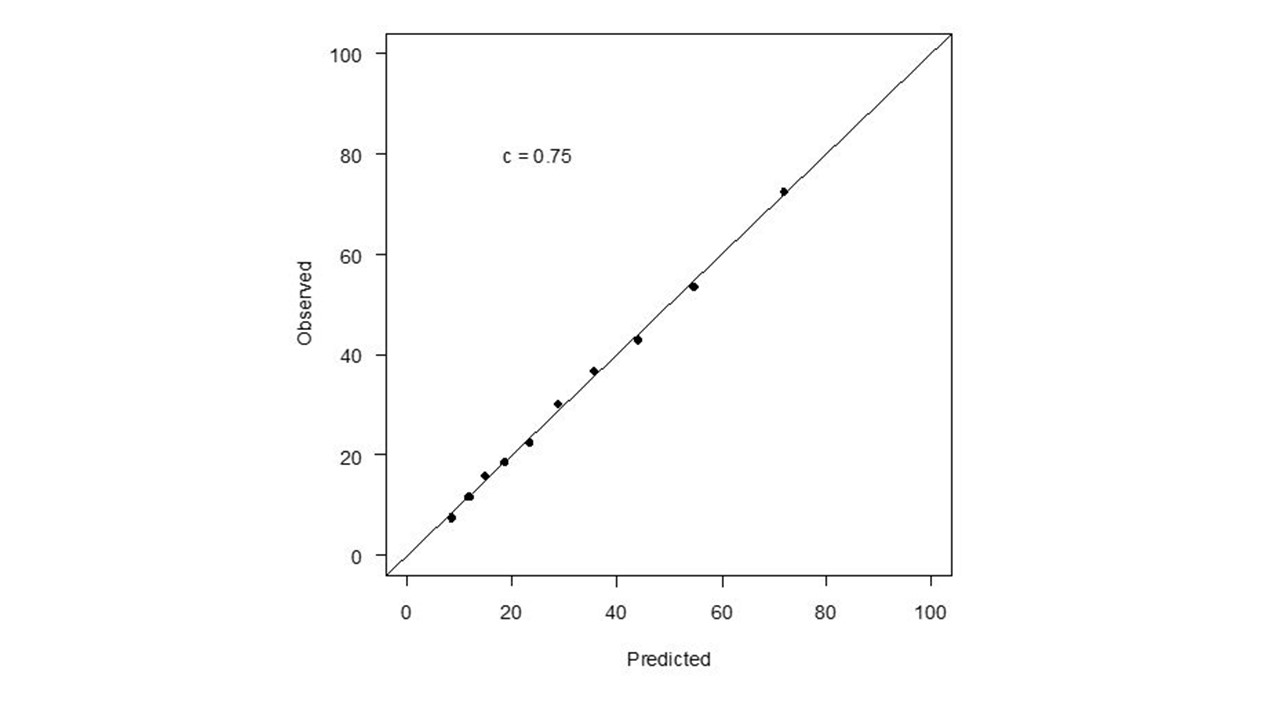

Supplement: S1 Fig — (JPG) [file pone.0275729.s001.jpg]
