# Supplementary material for: Derivation and validation of a predictive model for chronic stress in patients with cardiovascular disease
Source: PLoS One. 2022 Oct 18;17(10):e0275729. doi: 10.1371/journal.pone.0275729 (PMC9578618; doi:10.1371/journal.pone.0275729)
Supplement: S1 Table — (DOCX) [file pone.0275729.s002.docx]

**Supplementary Table 1.** Model coefficients for all factors in the final model to predict chronic stress in patients with cardiovascular disease.

| **Covariate** | **Coefficients** |
| --- | --- |
| Age | -0.027 *× age (in years)* |
| Female Sex | + 0.362 |
| Not currently working for pay | +0.404 |
| Just enough end of month finances | +0.684 |
| Not enough end of month finances | +1.095 |
| Avoiding care due to cost | +0.613 |
| ENRICHD social support instrument score | -0.083 |
| **y-intercept** | 1.336 |
