# Supplementary material for: Derivation and validation of a predictive model for chronic stress in patients with cardiovascular disease
Source: PLoS One. 2022 Oct 18;17(10):e0275729. doi: 10.1371/journal.pone.0275729 (PMC9578618; doi:10.1371/journal.pone.0275729)
Supplement: Attachment — Submitted filename: Response Letter v6.docx [file pone.0275729.s003.docx]

June 3^rd^, 2022

Richard Ibañez Dilla

*PLOS ONE*

Dear Mr. Ibañez Dilla

We are pleased to submit a revised version of our manuscript entitled *“Derivation and Validation of a Predictive Model for Chronic Stress in Patients with Cardiovascular Disease”.*

Thank you for considering our manuscript and I look forward to hearing from you.

Kim Smolderen

Please note: Below, we have copied the editor’s comments (in **bold**) and provided our responses in plain text. Sentences added to the manuscript in response to an editor or reviewer comment are indented and placed in *italic* font.

**Editor Comments:**

**Before we proceed with your manuscript, please address the following prompt in an updated Data Availability statement:**

**If there are ethical or legal restrictions on sharing a de-identified data set, please explain the restrictions in detail (e.g., data contain potentially identifying or sensitive patient information, data are owned by a third-party organization, etc.) and who has imposed them (e.g., a Research Ethics Committee or Institutional Review Board, etc.). Please provide non-author contact information for a data access committee, ethics committee, or other institutional body to which data requests may be sent. If applicable, please also provide any necessary information which interested researchers would need when requesting access to data in order to obtain the minimal data set for your study.**

**RESPONSE:** We have added the following sentence to our manuscript.

*Page 4 Line 3-5*

*” For the PORTRAIT study data requests can be sent to Yale University Institute Review Board, at* [*hrpp@yale.edu*](mailto:hrpp@yale.edu)*. For the TRIUMPH study data requests could be sent to the steering committee for the TRIUMPH registry at* [*dbuchanan@saint-lukes.org*](mailto:dbuchanan@saint-lukes.org)*.”*
